# Redefining Cardiac Involvement and Targets of Treatment in Systemic Immunoglobulin AL Amyloidosis

**DOI:** 10.1001/jamacardio.2024.2555

**Published:** 2024-08-21

**Authors:** Aldostefano Porcari, Ambra Masi, Ana Martinez-Naharro, Yousuf Razvi, Rishi Patel, Adam Ioannou, Muhammad U. Rauf, Giulio Sinigiani, Brendan Wisniowski, Stefano Filisetti, Jasmine Currie-Cathey, Sophie O’Beara, Tushar Kotecha, Dan Knight, James C. Moon, Gianfranco Sinagra, Ruta Virsinskaite, Janet Gilbertson, Lucia Venneri, Aviva Petrie, Helen Lachmann, Carol Whelan, Peter Kellman, Sriram Ravichandran, Oliver Cohen, Shameem Mahmood, Charlotte Manisty, Philip N. Hawkins, Julian D. Gillmore, Ashutosh D. Wechalekar, Marianna Fontana

**Affiliations:** 1 National Amyloidosis Centre, Division of Medicine, University College London, Royal Free Hospital, London, United Kingdom; 2 Centre for Diagnosis and Treatment of Cardiomyopathies, Cardiovascular Department, Azienda Sanitaria Universitaria Giuliano-Isontina (ASUGI), University of Trieste, Trieste, Italy; 3 European Reference Network for Rare, Low Prevalence and Complex Diseases of the Heart (ERN GUARD-HEART), Trieste, Italy; 4 Department of Cardio-Thoraco-Vascular Sciences and Public Health, University of Padua, Padua, Italy; 5 Cardiology University Department, Heart Failure Unit, IRCCS Policlinico San Donato, San Donato Milanese, Milan, Italy; 6 Faculty of Medicine, University of Milano, Milan, Italy; 7 Institute of Cardiovascular Science, University College London, London, United Kingdom; 8 Barts Heart Centre, West Smithfield, London, United Kingdom; 9 National Heart, Lung, and Blood Institute, National Institutes of Health, Bethesda, Maryland; 10 St Bartholomew’s Hospital, London, United Kingdom

## Abstract

**Importance:**

Cardiac amyloid infiltration is the key determinant of survival in systemic light-chain (AL) amyloidosis. Current guidelines recommend early switching therapy in patients with a nonoptimal or suboptimal response regardless of the extent of cardiac amyloid infiltration.

**Objective:**

To assess the differences between serum biomarkers, echocardiography, and cardiovascular magnetic resonance (CMR) with extracellular volume (ECV) mapping in characterizing cardiac amyloid, the independent prognostic role of these approaches, and the role of ECV mapping to guide treatment strategies.

**Design, Setting, and Participants:**

Consecutive patients newly diagnosed with systemic AL amyloidosis (2015-2021) underwent echocardiography, cardiac biomarkers, and CMR with ECV mapping at diagnosis. Data were analyzed from January to June 2024.

**Main Outcomes and Measures:**

The primary outcomes of the study were all-cause mortality and hematological response as defined according to validated criteria: no response (NR), partial response (PR), very good partial response (VGPR), and complete response (CR). Secondary outcomes were the depth and speed of hematological response and overall survival according to ECV.

**Results:**

Of 560 patients with AL amyloidosis, the median (IQR) age was 68 years (59-74 years); 346 patients were male (61.8%) and 214 female (38.2%). Over a median (IQR) 40.5 months 9-58 months), ECV was independently associated with mortality. In the landmark analysis at 1 month, long-term survival was independent of the achieved hematological response in ECV less than 0.30% and ECV of 0.31% to 0.40%, while it was dependent on the depth of the hematological response in ECV greater than 0.40%. In the landmark analysis at 6 months, survival was independent of the achieved hematological response in ECV less than 0.30% and dependent on achieving at least PR in ECV of 0.31% to 0.40%. Survival was dependent on achieving CR in ECV of 0.41% to 0.50% and ECV greater than 0.50%. Achieving a deep hematological response at 1 month was associated with better survival compared with 6 months in patients with ECV greater than 0.40% but not with ECV less than 0.40%.

**Conclusions and Relevance:**

This study found that ECV mapping, in systemic AL amyloidosis, is an independent predictor of prognosis, can help define the hematological response associated with better long-term outcomes for each patient and potentially inform treatment strategies.

## Key Points

**Question:**

What is the role of cardiovascular magnetic resonance (CMR) with extracellular volume (ECV) mapping in systemic light-chain (AL) amyloidosis?

**Findings:**

This cohort study involved 560 patients newly diagnosed with systemic AL amyloidosis undergoing CMR with ECV mapping before chemotherapy. Of the different approaches used to define cardiac involvement, ECV mapping was the only independent predictor of prognosis and provided information on the relation between the depth and rapidity of hematological response and outcomes, with a rapid and deep hematological response being important in patients with increasing ECV.

**Meaning:**

ECV mapping redefines the hematological response associated with better outcomes and has the potential to inform treatment strategies.

## Teaser

This cohort study analyzes data for 560 patients newly diagnosed with systemic AL amyloidosis to assess the differences between biomarkers, echocardiography, and cardiovascular magnetic resonance with extracellular volume mapping in characterizing cardiac amyloid infiltration.

## Introduction

Systemic immunoglobulin light-chain (AL) amyloidosis is characterized by the production of misfolded immunoglobulin light chains by an abnormal clone of plasma cells that subsequently deposits within the interstitial space of multiple organs, resulting in progressive organ dysfunction.^1^

Cardiac infiltration (cardiac AL amyloidosis, AL-CA), present in up to 80% of patients, is the main driver for mortality,^2^ with median survival ranging from 2 months to 4 years depending on cardiac disease severity.^3,4^ Serum cardiac biomarkers and echocardiography traditionally used to define the presence of cardiac involvement,^5,6,7^ although prognostically useful, do not directly measure cardiac amyloid infiltration.

Cardiovascular magnetic resonance (CMR) with late gadolinium enhancement (LGE) can visualize the continuum of cardiac amyloid infiltration; extracellular volume (ECV) provides a powerful quantitative measurement of the amyloid burden and is a strong independent predictor of mortality.^8^ Changes in ECV can track cardiac treatment response and correlate well with prognosis, even after adjusting for known predictors.^9,10^

Cytotoxic chemotherapy targets the plasma cells and suppresses light-chain production, with rapid and deep suppression being associated with improved outcomes.^1^ Current guidelines and practice recommend switching therapy in patients with a nonoptimal or suboptimal response at 1 or 3 months regardless of the presence and extent of cardiac amyloid infiltration.^11^

The aims of this study were to assess (1) the differences between serum biomarkers, echocardiography, and CMR with ECV mapping in characterizing cardiac involvement, (2) the independent prognostic role of serum biomarkers, echocardiography, and CMR with ECV mapping to predict prognosis, and (3) the potential of ECV mapping to guide treatment strategies.

## Methods

### Study Population

All patients were enrolled into a protocolized follow-up program at the National Amyloidosis Centre. Patients were treated in accordance with the Declaration of Helsinki and provided informed written consent for retrospective analysis and publication of their data with approval from the Royal Free Hospital ethics committee.

Study participants were individuals with systemic AL amyloidosis identified from a long-term prospective observational study of newly diagnosed patients (ALchemy) conducted at the National Amyloidosis Centre, United Kingdom (January 2015 to December 2021). All patients who underwent an echocardiogram, serum biomarker assessment, and CMR with ECV mapping at the time of diagnosis were eligible for inclusion. Individuals with a difference less than 20 mg/L between involved and uninvolved free light-chain (dFLC) at diagnosis were excluded because of a lack of validated response criteria.^12,13^ Patients who were treated with autologous stem cell transplant or treated at referring centers after assessment at the National Amyloidosis Centre (ie, lost at follow-up) were excluded (eFigure 1 in Supplement 1).

Before patients were enrolled, the diagnosis of AL amyloidosis was confirmed by central review of histological material inclusive of Congo red staining, with amyloid subtype being confirmed by immunohistochemistry with specific antibodies or mass spectrometry. All patients underwent comprehensive assessments, including electrocardiogram, echocardiography, N-terminal pro–B-type natriuretic peptide (NT-proBNP) measurements, and CMR with ECV mapping.

### Definition of Cardiac Involvement

Five approaches were used to define cardiac involvement:

- Historical criteria of AL-CA per expert consensus statements^5,14^ and randomized clinical trials: interventricular wall thickness greater than 12 mm in the absence of an alternative cause^5^ and/or NT-proBNP greater than 332 ng/L.^14,15,16^
- Validated “systemic AL score” by echocardiography^17^: 0 indicated AL-CA was unlikely; 1 to 4, possible AL-CA; and 5 or 6, typical AL-CA.
- NT-proBNP serum concentration^18^: less than 152 ng/L indicated no cardiac involvement.
- Mayo staging system: stage I is when cardiac troponin T and NT-proBNP values are both below the respective cutoffs (cardiac troponin T ≥0.035 ng/mL and NT-proBNP >332 ng/L); stage II, 1 value is elevated; and stage III, both are above the cutoff value. Mayo stage III was further subdivided using the European modification of Mayo 2004 staging, with Mayo stage III patients subdivided into IIIa (NT-proBNP <8500 ng/L) and IIIb (NT-proBNP ≥8500 ng/L).^19^
- ECV by CMR: no cardiac involvement when ECV is 0.30% or less; mild cardiac infiltration, ECV of 0.31% to 0.40%; moderate cardiac infiltration, ECV of 0.41% to 0.50%; and severe cardiac infiltration, ECV greater than 0.50%.^10^

### Echocardiography and CMR Image Acquisition and Analysis

Echocardiographic evaluation was performed using a GE Vivid E9 ultrasound machine equipped with a 5S probe. Measurements were performed offline using EchoPAC software (version 202) as previously described^10^ (Supplement 1).

All patients underwent CMR on a 1.5-T clinical scanner (Magnetom Aera; Siemens Healthcare). Within a conventional clinical scan (localizers and cine imaging with steady-state free precession [SSFP] sequence), LGE imaging was acquired with both magnitude inversion recovery and phase-sensitive inversion recovery sequence reconstructions with SSFP readouts. T1 measurement was performed with the use of the modified look-locker inversion recovery sequence.^20^ After a bolus of gadoterate meglumine (0.1 mmoL/kg, gadolinium-DOTA, Dotarem; Guerbet S.A. France) and LGE imaging, T1 mapping was repeated 15 minutes postcontrast using the same slice locations with the modified look-locker inversion recovery sequence, to produce automated inline ECV mapping reconstruction. For ECV measurement, the basal to mid segment of the interventricular septum was manually contoured at the endocardial border from the 4-chamber long axis map.^20^ All CMR image analysis was performed by assessors who were blinded to all other clinical and imaging data. The LGE pattern was classified into 3 groups according to phase-sensitive inversion recovery LGE transmurality: group 1, no LGE; group 2, subendocardial LGE only; and group 3, transmural LGE. Image analysis was performed offline using Osirix MD version 9.0 (Bernex). Information about the variability of ECV measurements was previously published.^10,21^

### Hematological Response and Mayo Staging System

Serum free light chain (FLC) measurements and serum immunofixation were performed at the National Amyloidosis Centre and assessed at baseline and monitored during follow-up. Hematological response at 1 month was defined according to validated International Society of Amyloidosis criteria (excluding urine immunofixation)^4,22,23^: no response (NR), less than 50% decrease in the difference in concentration between the aberrant vs uninvolved class of FLCs (dFLCs) from baseline; partial response (PR), 50% decrease in the dFLCs from baseline; very good partial response (VGPR), dFLCs less than 40 mg/L; and complete response (CR), the absence of a detectable monoclonal protein in serum with the normalization of the κ/λ ratio and a concentration of uninvolved FLCs greater than the involved-FLC concentration.^23^

### Study Outcome

The primary outcome of the study was all-cause mortality. All mortality data were obtained through the UK Office of National Statistics, which is the formal government registry for all deaths throughout the United Kingdom. The mortality end point was defined as time to death from baseline for all deceased patients and time to censor date (June 30, 2022) from baseline among the remaining patients.

### Statistical Analysis

Descriptive statistics in each of the study groups were calculated. Continuous variables were summarized by median (IQR) because data were not normally distributed according to the results of Kolmogorov-Smirnov test; categorical variables were summarized by frequencies and percentages. Differences between groups were evaluated using the Mann-Whitney test for continuous variables, while χ^2^ or Fisher exact tests were used for dichotomous variables. Survival was evaluated using Cox proportional hazard regression analysis, providing estimated hazard ratios (HRs) with 95% CIs and Kaplan-Meier curves. Landmark analyses at 1 month and 6 months after the initiation of chemotherapy were performed to assess the impact of depth and rapidity of the hematological response on long-term survival among patients with systemic AL amyloidosis, stratified by ECV value. Only those patients who survived until the designated landmark time were included in the analysis.

The 5 different approaches used to define cardiac involvement were selected a priori based on clinical relevance: historical definition of CA (using wall thickness and NT-proBNP), echocardiographic systemic AL score, NT-proBNP value with a single cutoff, Mayo staging system (using NT-proBNP and troponin), and ECV mapping by CMR. The proportional hazards assumption was checked and confirmed. The 5 approaches were first explored with univariable Cox regression analysis, and variables that were statistically significant predictors of outcome (*P* ≤ .10) were entered into a multivariable Cox proportional hazards analysis to determine which covariates were independent predictors of mortality. Possible collinearity among candidate predictors was assessed using variance inflation factors with a threshold equal to 5.

All statistical analyses were performed using SPSS Statistics 24.0 package (IBM) version 20 and Stata release 15 (StataCorp). We defined a *P* value <.05 as statistically significant. Data were analyzed from January to June 2024.

## Results

Five-hundred sixty patients with newly diagnosed AL amyloidosis were included. All patients received bortezomib-based first-line treatment. Baseline characteristics of the study population are showed in the Table and eTable 1 in Supplement 1.

**Table.** Baseline Characteristics of the Study Population

Characteristic	All (N = 560), No. (%)
Age, median (IQR), y	68 (59 to 74)
Sex	
Male	346 (61.8)
Female	214 (38.2)
SBP, median (IQR), mm Hg	115 (105 to 130)
Ischemic heart disease	57 (10.2)
Diabetes	56 (10)
Hypertension	156 (27.9)
Atrial fibrillation	70 (12.5)
eGFR, mL/min	71 (55 to 90)
NYHA class	
I	95 (19.4)
II	301 (61.4)
III	79 (16.1)
IV	15 (3.1)
NT-proBNP, median (IQR), ng/L	2148 (518 to 5751)
Troponin T, median (IQR), ng/mL	50 (24 to 106)
κ/λ Ratio, median (IQR)	8 (1.3 to 23.3)
VGPR or CR at 1 mo	167 (29.8)
IVS, median (IQR), mm	14 (11 to 16)
RWT, median (IQR)	0.62 (0.51 to 0.77)
E/e′ ratio, median (IQR)	12.7 (9.2 to 18.2)
LVEF, median (IQR), %	58 (51 to 62)
GLS, median (IQR), %	−14.5 (−19.1 to −10.5)
ECV, median (IQR), %	0.45 (0.36 to 0.53)
β-Blockers	134 (23.9)
ACEi/ARBs	169 (30.2)
Loop diuretic	286 (51.1)
MRAs	64 (11.4)

Abbreviations: ACEi, angiotensin-converting enzyme inhibitors; ARBs, angiotensin receptor blockers; CR, complete response; ECV, extracellular volume; E/e′, ratio of E-wave velocity to peak e′ velocity; eGFR, estimated glomerular filtration rate; GLS, global longitudinal strain; IVS, interventricular septum thickness; LVEF, left ventricular ejection fraction; MRAs, mineralocorticoid receptor antagonists; NT-proBNP, N-terminal pro–B-type natriuretic peptide; NYHA, New York Heart Association; RWT, relative wall thickness; SBP, systolic blood pressure; VGPR, very good partial response.

The median (IQR) age was 68 years (59-74 years); 346 patients were male (61.8%) and 214 female (38.2%). NYHA class was I in 95 patients (19.4%), II in 301 patients (61.4%), III in 79 patients (16.1%), and IV in 15 patients (3%). Median (IQR) eGFR was 71 mL/min (55-90 mL/min); median (IQR) NT-proBNP was 2148 ng/L (518-5751 ng/L). On CMR imaging, median (IQR) ECV was 0.45% (0.36%-0.53%).

Hematological response at 1 month after initiation of chemotherapy was as follows: NR in 238 patients (42.5%), PR in 155 patients (27.7%), VGPR in 144 patients (25.7%), and CR in 23 patients (4.1%). Hematological response at 6 months after initiation of chemotherapy was NR in 55 patients (19.4%), PR in 83 patients (29.2%), VGPR in 92 patients (32.4%), and CR in 54 patients (19.0%).

### Prevalence of Cardiac Involvement According to Different Definitions

The prevalence of the presence of cardiac involvement using the 4 approaches based on cardiac biomarkers, echocardiography, or their combination was assessed across the range of amyloid infiltration as measured by ECV mapping (eFigure 2 in Supplement 1).

Among patients fulfilling the criteria for cardiac involvement, CMR with ECV mapping confirmed cardiac amyloid infiltration in most cases but found 27 (6%) to 37 (16%) patients without cardiac involvement (ECV ≤0.30%). Conversely, among patients not fulfilling the criteria, CMR with ECV mapping identified cardiac amyloid infiltration in 17 patients (41.5%) (based on NT-proBNP) to 49 patients (55.7%) (based on historical criteria).

### Serum Biomarkers, Echocardiography, CMR With ECV Mapping, and Survival

During a median (IQR) follow-up of 40.5 months (9-58 months), 211 patients (37.6%) died. In all multivariable Cox regression models, after adjusting for age and NYHA class, only ECV remained an independent predictor of survival (eTables 2-4 in Supplement 1). Kaplan-Meier curves for different ECV brackets are shown in eFigure 3 in Supplement 1. Patients with an ECV of 0.30% or less (no cardiac amyloid infiltration) had significantly better survival than patients with higher ECV, with higher mortality rates being associated with increasing degrees of cardiac amyloid infiltration (eFigure 3 in Supplement 1).

### Depth of Hematological Response and Overall Survival According to ECV

#### 1-Month Landmark Cohort Analysis

**Figure 1.**
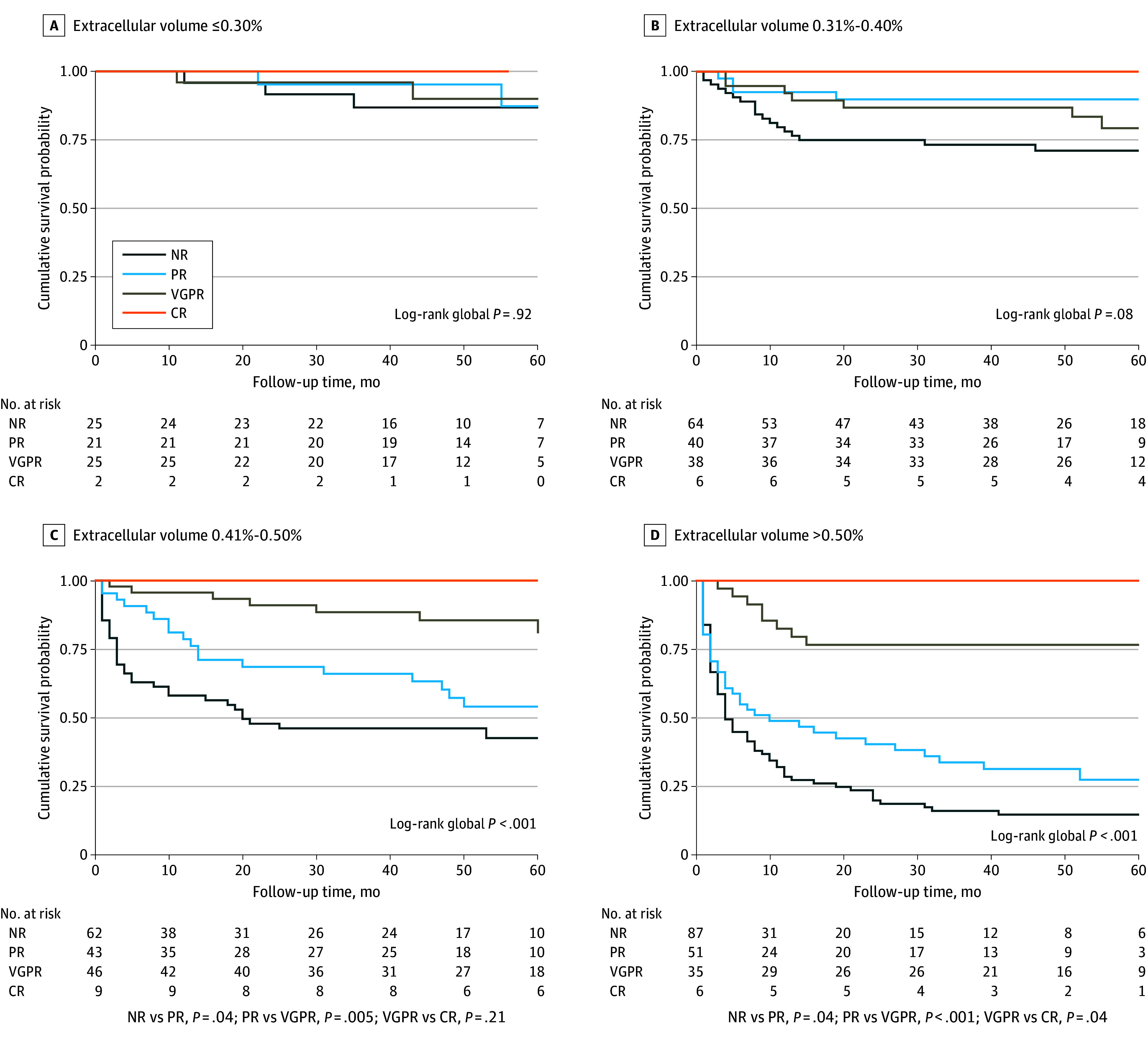
Association of Early Response (at 1 Month) and Survival Stratified by Baseline Extracellular Volume Kaplan-Meier analysis of 60-month survival for patients with AL amyloidosis according to hematological response at 1 month. *P* values for intergroup comparison are shown only for subgroups with global *P* < .05. CR indicates complete response; NR, no response; PR, partial response; VGPR, very good partial response.

Patients with ECV of 0.30% or less (no cardiac involvement) had no difference in long-term survival regardless of the hematological response (Figure 1). In patients with ECV of 0.31% to 0.40% (early AL-CA), those achieving NR demonstrated poorer survival. Patients with ECV of 0.41% to 0.50% had long-term survival when achieving CR or VGPR and lower survival when achieving PR, with the poorest survival with NR (Figure 1). In patients with ECV greater than 0.50%, the degree of response was associated with survival, with good long-term survival only when achieving a CR and worse survival with VGPR, PR, and NR (Figure 1).

Conversely, when using the Mayo stage, better survival was associated only with patients achieving at least VGPR at 1 month in all Mayo stages (eFigure 4 in Supplement 1).

#### 6-Month Landmark Cohort Analysis

Patients with ECV of 0.30% or less (no cardiac involvement) had good long-term survival regardless of the hematological response (eFigure 5 in Supplement 1). Patients with ECV of 0.31% to 0.40% achieving CR, VGPR, or PR had similar long-term survival, with only NR associated with poorer survival.

Patients with ECV of 0.41% to 0.50% and ECV greater than 0.50% had good long-term survival only if achieving CR with declining survival with VGPR, PR, and NR, with the poorest survival with NR (eFigure 5 in Supplement 1). In all multivariable models for the landmark analysis at 6 months, after adjusting for age and NYHA class, ECV remained an independent predictor of survival (eTables 2-4 in Supplement 1).

Figure 1 and eFigure 5 in Supplement 1 show patients’ overall survival in the 1-month and 6-month landmark cohorts, stratified by their ECV value and hematological response.

### Speed of Hematological Response and Overall Survival According to ECV

Because a deep hematological response (defined as ≥VGPR) at 1 month after treatment was associated with outcomes, we analyzed the survival of patients achieving an early deep response (at 1 month) and compared them with those who had not achieved this response at 1 month but had reached a deep response later (at 6 months). There were 443 evaluable patients with response data at 1 and 6 months. Two hundred eighty-five patients (63.9%) had not achieved a CR or VGPR at 1 month. Of them, 146 patients (32.9%) improved their response to CR or VGPR at 6 months.

**Figure 2.**
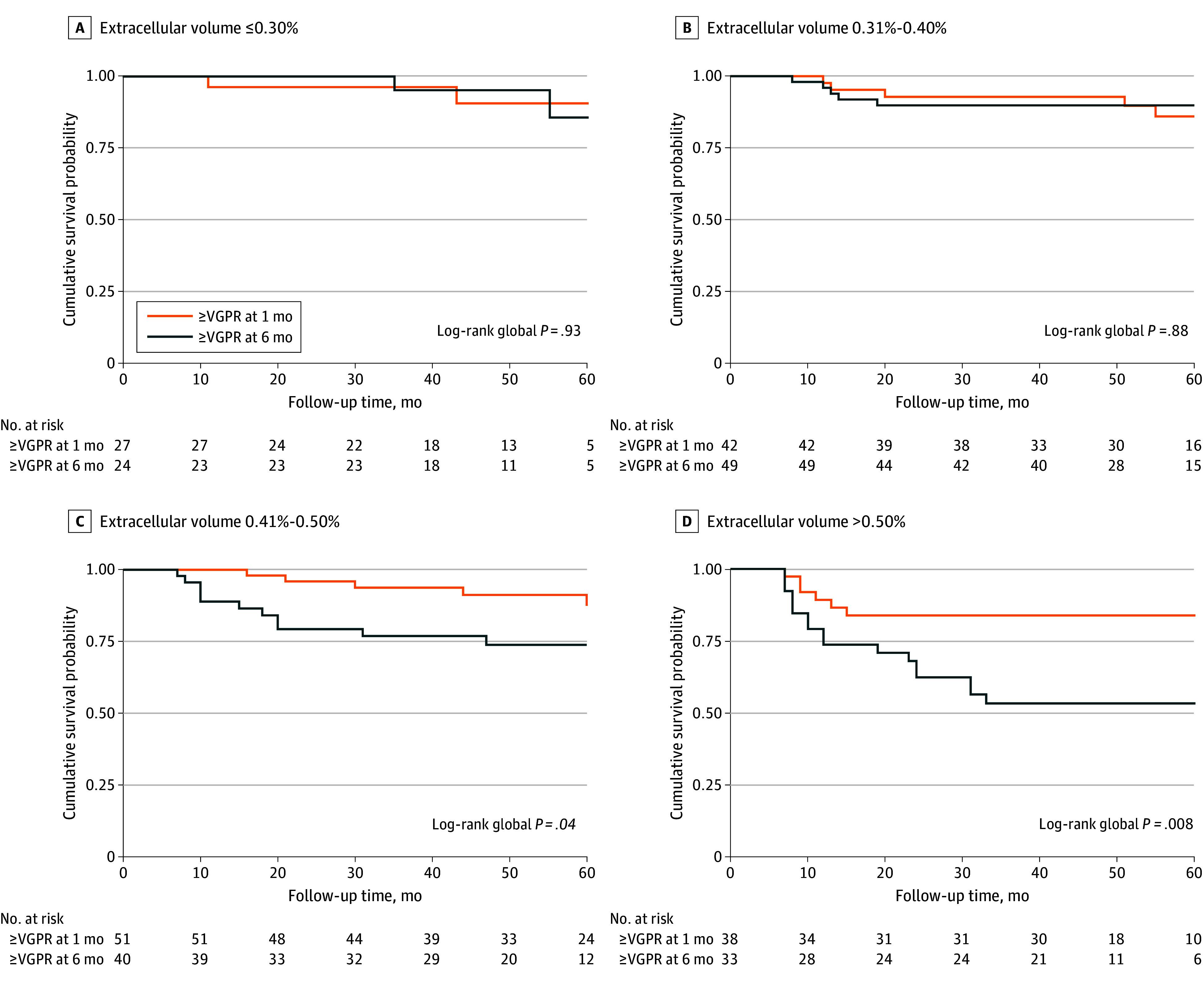
Association of Early Deep Hematological Response (Very Good Partial Response or Better [≥VGPR] at 1 Month) vs Late Deep Hematological Response (≥VGPR at 6 Months) and Overall Survival Stratified by Baseline Extracellular Volume Kaplan-Meier analysis of 60-month survival for patients with AL amyloidosis according to time to a hematological response ≥VGPR. *P* values for intergroup comparison are shown only for subgroups with global *P* < .05.

Long-term survival was similar among patients with ECV of 0.30% or less (no cardiac involvement) and with ECV of 0.31% to 0.40% regardless of achieving early (≥VGPR at 1 month) vs late (≥VGPR at 6 months) deep hematological response (Figure 2). In patients with ECV of 0.41% to 0.50% and with ECV greater than 0.50%, better long-term survival was observed only among patients achieving early deep hematological response. Figure 2 shows patients’ overall survival according to the speed of deep hematological response, stratified by their ECV value.

## Discussion

This prospective study demonstrates the ability of CMR with ECV mapping in patients with systemic AL amyloidosis to characterize the degree of cardiac involvement, predict long-term outcomes, and inform management strategies. Our study demonstrated the following 3 findings. First, the different approaches currently used to define the presence and severity of cardiac involvement, which included serum biomarkers, echocardiography, and CMR with ECV mapping, produce widely different results. Second, when all approaches currently used to define cardiac involvement were assessed in multivariable models, ECV mapping was the only independent predictor of prognosis. Third, the degree of cardiac infiltration, as measured by ECV mapping, provided information on the effect of the depth and rapidity of hematological response required; the key finding was that not all patients required a rapid hematological response, but a rapid and deep hematological response was crucial in patients with increasing degrees of cardiac infiltration, as redefined by CMR with ECV mapping. These findings have the potential to inform early switching therapy according to hematological response and pretreatment ECV to improve survival.

Blood biomarkers are heavily influenced by both cardiac and noncardiac factors, such as fluid status, neurohormonal activation, atrial fibrillation, body mass index (adiposity), and kidney function, and therefore do not necessarily represent the degree of cardiac amyloid infiltration.^10^ Approaches based on the structural and functional myocardial changes, such as the degree of ventricular thickness alone or in combination with functional changes, are influenced by comorbidities common in these patients, such as hypertension, valvular heart disease, or kidney failure. CMR with gadolinium contrast, by isolating the signal from the ECV, provides an accurate measure of myocardial amyloid deposition as a global increase in ECV most likely represents an increase in the amyloid burden. The ability of ECV mapping to stratify prognosis and independently predict outcomes across various degrees of infiltration, ranging from no amyloid deposits (ECV ≤0.30%) to severe disease (ECV >0.50%), provides a significant advantage in assessing myocardial amyloid load over both cardiac biomarkers and echocardiography.^10^

Achieving a deep response to chemotherapy has long been the goal of treatment in all patients with systemic AL amyloidosis irrespective of the presence and degree of cardiac involvement, with early deep response (ie, within 1 month of treatment initiation) associated with improved survival across all disease stages defined using the Mayo stage system^23^ as opposed to achieving a deep response later. We reported an algorithm to allow for treatment switching as early as 1 month.^24^ The current guidelines and practice recommend early switching therapy in patients with a nonoptimal or suboptimal response at 1 or 3 months. However, switching therapy too early may mean the true benefit is not obtained and potentially well-tolerated therapies are abandoned for second-line regimens. Furthermore, the increasing cost of therapy means that decisions also have a health-economic angle in most health care systems with limited access to therapies. While the current findings do not contradict the need for a deep response to treatment, the key finding is that patients with mild or no cardiac amyloid deposition (ECV <0.40%) had similar outcomes regardless of the timing of the response, compared with patients with moderate to advanced cardiac involvement (ECV >0.40%), where a VGPR or better at 1 month markedly improved survival. These findings underscore the importance of pretreatment CMR with ECV mapping in redefining goals of chemotherapy at an individual level and informing a shift from universally rapid response in all patients to individualized approaches based on presence and degree of cardiac amyloid infiltration (eTable 5 in Supplement 1). Survival in patients with AL amyloidosis is closely dependent on the ability to achieve a satisfactory hematological response rather than on the specific chemotherapy regimen adopted to achieve that hematological response. To improve survival, at least a VGPR is required at 1 month and CR at 6 months in patients with moderate infiltration (ECV 0.41%-0.50%), while in patients with severe infiltration (ECV >0.50%), the goal is even more stringent, that is, CR at 1 month to improve outcomes.

Native myocardial T1 is closely related to ECV, and changes in response to treatment have been recently associated with mortality.^25^ Native myocardial T1 has the significant advantage of being measured with a single image acquisition (reduced acquisition time) without the need for contrast (relevant in patients with kidney disease). The identification of accurate cutoff values of native myocardial T1 associated with increased ECV to redefine cardiac involvement among patients with systemic AL amyloidosis should be explored in future studies.

CMR plays a pivotal role in guiding these individualized treatment strategies, advocating for careful consideration before switching therapies early to maximize the benefit from first-line treatments (commonly well tolerated), especially in patients with no or mild cardiac infiltration. Conversely, chemotherapy regimens with highly likelihood of early CR or early addition of novel agents should be considered in patients with significant cardiac infiltration. This is a complex clinical challenge to address in practice because the patients with advanced disease tolerate treatment poorly, are at high risk of treatment toxicity, and hence receive gentle reduced-dose treatment regimens, compared with patients with early-stage disease, where tolerance is excellent and intensive rapidly effective regimens are typically chosen.

### Limitations

The limitations of this study performed at a third-level referral Centre for amyloidosis include its longitudinal, single-center nature. Patients with contraindication to contrast CMR study were excluded (ie, advanced kidney impairment, permanent pacemaker or other cardiac devices, difficulties in lying flat). In addition, the absence of serial ECV measurements highlights the need for external validation in a broader cohort.

## Conclusions

This study found that CMR with ECV mapping has the key advantage of accurately characterizing the presence and extent of cardiac amyloid infiltration compared with both serum cardiac biomarkers and echocardiography. ECV mapping can independently predict overall survival in systemic AL amyloidosis and has the ability to help define hematological response associated with better long-term survival according to the presence and severity of cardiac amyloid infiltration.

## Notes

Supplement 1.
eMethods
**eTable 1. **Baseline characteristics of the study population according to definitions of cardiac involvement **eTable 2. **Univariable and 4 multivariable Cox regression analyses for all-cause mortality according to different criteria of cardiac involvement **eTable 3. **Univariable and 4 multivariable Cox regression analyses for all-cause mortality according to different criteria of cardiac involvement **eTable 4. **Multivariable Cox regression analyses for all-cause mortality according to different criteria of cardiac involvement in the landmark cohort at 6 months **eTable 5. **Proposal for a new Staging System to redefine cardiac involvement in systemic AL amyloidosis **eFigure 1. **Diagram of the study **eFigure 2. **Distribution of cardiac amyloid load measured by ECV values using different criteria of cardiac involvement **eFigure 3. **Kaplan-Meier analysis demonstrating the impact of baseline ECV mapping on overall survival among patients with AL amyloidosis **eFigure 4. **Association of early response (1-month) and survival stratified by baseline Mayo stages **eFigure 5. **Association of deep response at 6 months and survival stratified by baseline ECV in AL patients with NR/PR at 1 month

Supplement 2. Data sharing statement
