# Supplementary material for: Redefining Cardiac Involvement and Targets of Treatment in Systemic Immunoglobulin AL Amyloidosis
Source: JAMA Cardiol. 2024 Aug 21;9(11):982–9. doi: 10.1001/jamacardio.2024.2555 (PMC11339700; doi:10.1001/jamacardio.2024.2555)
Supplement: Supplement 1. — eMethods eTable 1. Baseline characteristics of the study population according to definitions of cardiac involvement eTable 2. Univariable and 4 multivariable Cox regression analyses for all-cause mortality according to different criteria of cardiac involvement eTable 3. Univariable and 4 multivariable Cox regression analyses for all-cause mortality according to different criteria of cardiac involvement eTable 4. Multivariable Cox regression analyses for all-cause mortality according to different criteria of cardiac involvement in the landmark cohort at 6 months eTable 5. Proposal for a new Staging System to redefine cardiac involvement in systemic AL amyloidosis eFigure 1. Diagram of the study eFigure 2. Distribution of cardiac amyloid load measured by ECV values using different criteria of cardiac involvement eFigure 3. Kaplan-Meier analysis demonstrating the impact of baseline ECV mapping on overall survival among patients with AL amyloidosis eFigure 4. Association of early response (1-month) and survival stratified by baseline Mayo stages eFigure 5. Association of deep response at 6 months and survival stratified by baseline ECV in AL patients with NR/PR at 1 month [file jamacardiol-e242555-s001.pdf]

Supplemental Online Content

Porcari A, Masi A, Martinez-Naharro A, et al. Redefining cardiac involvement and targets of treatment in systemic immunoglobulin AL amyloidosis. *JAMA Cardiol*. Published online August 21, 2024. doi:10.1001/jamacardio.2024.2555

eMethods
eTable 1. Baseline characteristics of the study population according to definitions of cardiac involvement
eTable 2. Univariable and 4 multivariable Cox regression analyses for all-cause mortality according to different criteria of cardiac involvement
eTable 3. Univariable and 4 multivariable Cox regression analyses for all-cause mortality according to different criteria of cardiac involvement
eTable 4. Multivariable Cox regression analyses for all-cause mortality according to different criteria of cardiac involvement in the landmark cohort at 6 months
eTable 5. Proposal for a new Staging System to redefine cardiac involvement in systemic AL amyloidosis
eFigure 1. Diagram of the study
eFigure 2. Distribution of cardiac amyloid load measured by ECV values using different criteria of cardiac involvement
eFigure 3. Kaplan-Meier analysis demonstrating the impact of baseline ECV mapping on overall survival among patients with AL amyloidosis
eFigure 4. Association of early response (1-month) and survival stratified by baseline Mayo stages
eFigure 5. Association of deep response at 6 months and survival stratified by baseline ECV in AL patients with NR/PR at 1 month

This supplemental material has been provided by the authors to give readers additional information about their work.

© 2024 Porcari A et al. *JAMA Cardiology.*

eMethods

Echocardiography acquisition and analysis

At least three consecutive beats were recorded for each view, and images were stored for off-line analysis. Left ventricular (LV) chamber morphology was assessed following the latest American Society of Echocardiography/European Association of Cardiovascular Imaging Guideline[19]. LV mass and LV mass index were calculated using Devereux's formula and RWT was calculated as: 2 × posterior wall thickness in diastole/LVEDD (2 × PWTd/LVEDD). Left atrial area and right atrial area were measured in the four-chamber view. LV ejection fraction (LVEF) was calculated with the biplane Simpson's method from volumes acquired in both the four-chamber and the two-chamber views. Lateral mitral annular plane systolic excursion (MAPSE) and tricuspid annular plane systolic excursion (TAPSE) were assessed with M-mode in the four-chamber view. LV early (E wave) and late (A-wave) diastolic filling, its ratio (E/A), and the E-wave deceleration time (DT) were evaluated with pulsed Doppler in the 4-chamber view. Lateral and septal mitral annulus velocities (e′ wave) were assessed with tissue Doppler in the four-chamber view; the ratio between the LV early diastolic filling wave and lateral mitral annulus velocity (E/e′) was calculated[20]. Digitally acquired clips were considered suitable for offline 2D speckle strain imaging analysis if at least three cardiac cycles were available, with high frame rates (70 to 100 frame/s) and without dropout of more than one LV segment or significant foreshortening of the ventricle. The endocardial border was traced at the end-diastolic frame in the apical view. End-diastole was defined by the QRS complex or by the frame just before mitral valve closure. The software tracked speckles along the endocardial and epicardial borders throughout the cardiac cycle, and the width of the region of interest was adjusted to fit the entire myocardium. All strain and strain-derived variables were measured in the apical four-chamber view. Peak longitudinal strain was computed automatically, generating regional data from six segments (basal, mid, apical inter- ventricular septum and basal, mid, apical lateral wall), to calculate an average value. All the echocardiogram analysis was performed blinded to CMR results.

© 2024 Porcari A et al. *JAMA Cardiology.*

eTable 1. Baseline characteristics of the study population according to definitions of cardiac involvement

Parameters	All (n=560)	HC – (n=88)	HC + (n=472)	MS I (n=100)	MS II (n=197)	MS IIIa (n=180)	MS IIIb (n=83)	AL Score <1 (n=61)	AL score 1-4 (n=229)	AL score >4 (n=270)
Age, y	68 [59-74]	64 [55-71]	68 [60-74]	64 [56-72]	68.5 [59-74]	69 [62-74]	67.5 [57-74]	68 [58-74]	69 [60-74]	67.5 [59-74]
Male, %	61.8% (346)	47.7 (42)	64.4 (304)	51% (51)	54.8% (108)	71.1% (128)	71.1% (59)	59% (36)	58.5% (134)	65.2% (176)
SBP, mmHg	115 [105-130]	127 [110-137]	115 [104-126]	127 [112-138]	119 [107-130]	115 [104-125]	106 [97-116]	130 [115-138]	120 [107-134]	110 [101-121]
Ischemic heart disease	10.2% (57)	8% (7)	10.6% (50)	11% (11)	7.6% (15)	11.1% (20)	13.3% (11)	4.9% (3)	10% (23)	11.5% (31)
Diabetes	10% (56)	14.8% (13)	9.1% (43)	14% (14)	5.6% (11)	13.9% (25)	7.2% (6)	14.8% (9)	9.2% (21)	9.6% (26)
Hypertension	27.9% (156)	25% (22)	28.4% (134)	26% (26)	26.4% (52)	33.3% (60)	21.7% (18)	37.7% (23)	29.7% (68)	24.1% (65)
Atrial fibrillation	12.5% (70)	4.5% (4)	14% (66)	4% (4)	13.2% (26)	16.1% (29)	13.3% (11)	1.6% (1)	9.6% (22)	17.4% (47)
eGFR	71 [55-90]	87 [66-90]	69 [53-88]	86 [66-90]	76 [60-90]	67 [54-85]	57 [41-76]	79 [61-90]	73 [54-90]	70 [53-88]
NYHA class										
I	19.4% (95)	83.8% (31)	14.1% (64)	81.4% (35)	28.1% (52)	3.9% (7)	1.2% (1)	70.7% (29)	29.2% (54)	4.5% (12))
II	61.4% (301)	13.5% (5)	65.3% (296)	16.3% (7)	67% (124)	78.8% (141)	34.9% (29)	29.3% (12)	61.6% (114)	66.3% (175)
III	16.1% (79)	0%	17.4% (79)	2.3% (1)	4.3% (8)	16.2% (29)	49.4% (41)	0% (0)	8.1% (15)	24.2% (64)
IV	3.1% (15)	2.7% (1)	3.1% (14)	0% (0)	0.5% (1)	1.1% (2)	14.5% (12)	0% (0)	1.1% (2)	4.9% (13)
NT-proBNP	2148 [518-5751]	193 [119-259]	2894 [984-6783]	198 [117-260]	1236 [584-2474]	3730 [1895-5753]	12767 [9874-20834]	255 [135-524]	894 [321-2410]	4974 [2416-8691]
TnT	50 [24-106]	13 [8-23]	61 [35-123]	13 [8-21]	35 [22-42]	97 [68-141]	152 [98-219]	17 [9-27]	34 [21-62]	82 [49-143]
k/l ratio	8.0 [1.3-23.3]	4.2 [0.2-15.2]	8.6 [1.7-24.8]	2.4 [0.1-12.7]	7.8 [1.6-20.8]	10.1 [2.7-29.0]	13.3 [2.4-28.0]	3.7 [0.2-15.8]	5.2 [0.4-17.6]	11.0 [3.2-28.6]
VGPR or CR at 1 mo	29.8% (167)	36.4% (32)	28.6% (135)	36% (36)	32% (63)	26.7% (48)	24.1% (20)	34.4% (21)	32.3% (74)	26.7% (72)
IVS, mm	14 [11-16]	10 [9-11]	14 [12-16]	11 [10-12]	13 [11-15]	15 [13-16]	16 [14-17]	10 [9-12]	12 [11-14]	15 [14-16]
RWT	0.62 [0.51-0.77]	0.46 [0.40-0.53]	0.65 [0.55-0.80]	0.48 [0.41-0.56]	0.58 [0.51-0.69]	0.68 [0.59-0.81]	0.75 [0.59-0.85]	0.44 [0.39-0.50]	0.55 [0.48-0.64]	0.71 [0.62-0.84]
E/E'	12.7 [9.2-18.2]	8.5 [6.8-10.3]	14 [11-20]	9 [7-12]	11 [9-16]	16 [12-21]	18 [14-24]	8.3 [7-10]	10 [8-13]	17 [14-22]
LVEF, %	58 [51-62]	61 [58-65]	57 [49-62]	61 [56-65]	60 [55-64]	56 [48-62]	53 [43-56]	60 [55-65]	60 [56-65]	54 [46-60]
GLS, %	-14.5 [-19.1 to -10.5]	-20.8 [-22.9 to -18.8]	-13.0 [-17.0 to -10.0]	-20.2 [-22.2 to -17.0]	-16.0 [-19.2 to -12.9]	-12.0 [-15.6 to -9.6]	-10.1 [-12.3 to -7.6]	-20.9 [-23.5 to -18.2]	-17.3 [-20.5 to -14.6]	-11.0 [-13.3 to -8.7]

© 2024 Porcari A et al. *JAMA Cardiology.*

**ECV, %**	0.45 [0.36-0.53]	0.31 [0.28-0.36]	0.48 [0.39-0.54]	0.31 [0.28-0.36]	0.41 [0.34-0.49]	0.49 [0.43-0.54]	0.56 [0.49-0.60]	0.30 [0.28-0.33]	0.39 [0.33-0.46]	0.51 [0.46-0.57]
**Beta blockers**	23.9% (134)	9.1% (8)	26.7% (126)	11% (11)	24.4% (48)	23.3% (42)	39.8% (33)	9.8% (6)	18.3% (42)	31.9% (86)
**ACEi/ARBs**	30.2% (169)	34.1% (30)	29.4% (139)	34% (34)	33% (65)	26.7% (48)	26.5% (22)	44.3% (27)	30.6% (70)	26.7% (72)
**Loop diuretic**	51.1% (286)	33% (29)	54.4% (257)	31% (31)	40.6% (80)	64.4% (116)	71.1% (59)	41% (25)	41.9% (96)	61.1% (165)
**MRAs**	11.4% (64)	6.8% (6)	12.3% (58)	6% (6)	8.6% (17)	12.8% (23)	21.7% (18)	1.6% (1)	9.2% (21)	15.6% (42)

Entries are median IQR for numerical variables and percentage (frequency) for categorical variables

ACEi, angiotensin-converting enzyme inhibitors; AL, light chain; ARBs, angiotensin receptor blockers; CR, complete response; ECV, extracellular volume; eGFR, estimated glomerular filtration rate; GLS, global longitudinal strain; HC, traditional criteria of cardiac involvement; HR, haematological response; IVS, interventricular septum thickness; LVEF, left ventricular ejection fraction; MRAs, mineralocorticoid receptor antagonists; MS, Mayo staging system; NT-proBNP, N-terminal pro-B-type natriuretic peptide; NYHA, New York Heart Association; RWT, relative wall thickness; SBP, systolic blood pressure; TnT, troponin T, VGPR, very good partial response.

© 2024 Porcari A et al. *JAMA Cardiology.*

eTable 2. Univariable and 4 multivariable Cox regression analyses for all-cause mortality according to different criteria of cardiac involvement.

**Variables**	**Univariable**		**Multivariable A** (including as covariates all parameters except for the Mayo staging system and NT-proBNP <152 ng/L)		**Multivariable B** (including as covariates all parameters except for the systemic AL score and NT-proBNP <152 ng/L)		**Multivariable C** (including as covariates all parameters except for the systemic AL score and the Mayo staging system)		**Multivariable D** (including as covariates all parameters except for the systemic AL score, the Mayo staging system and historical criteria)	
	**HR (95% CI)**	**p value**	**HR (95% CI)**	**p value**	**HR (95% CI)**	**p value**	**HR (95% CI)**	**p value**	**HR (95% CI)**	**p value**
Age, per year	1.018 (1.004–1.031)	**0.010**	1.02 (1.01-1.04)	**0.001**	1.02 (1.008-1.03)	**0.002**	1.02 (1.01-1.038)	**0.001**	1.02 (1.01-1.04)	**0.001**
NYHA class	Reference		Reference		Reference		Reference		Reference	
II	4.07 (2.19-7.55)	**<0.001**	2.43 (1.21-4.87)	**0.012**	2.63 (1.27-5.46)	**0.009**	2.68 (1.32-5.46)	**0.006**	2.46 (1.26-4.82)	**0.008**
III	13.94 (7.33-26.50)	**<0.001**	7.17 (3.42- 15.02)	**<0.001**	7.32 (3.33- 16.10)	**<0.001**	8.11 (3.83-17.15)	**<0.001**	7.45 (3.66-15.15)	**<0.001**
IV	36.10 (16.38-79-70)	**<0.001**	14.35 (5.92-34.78)	**<0.001**	13.59 (5.23-35.25)	**<0.001**	15.34 (6.34-37.11)	**<0.001**	14.22 (5.98-33.88)	**<0.001**
Historical Criteria	5.29 (2.71-10.32)	**<0.001**					0.92 (0.35-2.45)	0.87		
NT-proBNP <152 ng/L	0.09 (0.02-0.39)	**<0.001**							0 (0-0)	0.95
Mayo Stage	Reference				Reference					
II	2.33 (1.24-4.38)	**0.008**			0.70 (0.30-1.64)	0.42				
IIIa	5.30 (2.90-9.70)	**<0.001**			0.90 (0.38-2.14)	0.82				
IIIb	12.03 (6.47-22.40)	**<0.001**			0.95 (0.39-2.39)	0.93				
"Systemic AL score"	Reference		Reference							
Possible	3.02 (1.30-6.98)	**0.010**	1.14 (0.43-2.98)	0.78						
Typical	7.83 (3.46-17.71)	**<0.001**	1.41 (0.53-3.76)	0.49						
ECV per each 10%	2.25 (1.90-2.65)	**<0.001**	1.46 (1.24-1.73)	**<0.001**	1.50 (1.28-1.75)	**<0.001**	1.52 (1.31 – 1.78)	**<0.001**	1.52 (1.30-1.77)	**<0.001**

In order to avoid statistical coupling of variables, separate multivariable models were performed that excluded coupled parameters (e.g., four separate models were created for systemic AL score, Mayo staging system, NT-proBNP <152 ng/L and historical criteria). For all the variables, the variable inflation factor was <2 suggesting that multicollinearity was not an issue.

AL, Light Chain; CI, Confidence Interval; ECV, Extracellular Volume; HR, Hazard Ratio; NYHA, New York Heart Association.

© 2024 Porcari A et al. *JAMA Cardiology.*

eTable 3. Univariable and 4 multivariable Cox regression analyses for all-cause mortality according to different criteria of cardiac involvement

**Variables**	**Univariable**		**Multivariable A** (including as covariates all parameters except for the Mayo staging system and NT-proBNP <152 ng/L)		**Multivariable B** (including as covariates all parameters except for the systemic AL score, NT-proBNP <152 ng/L and traditional criteria)		**Multivariable C** (including as covariates all parameters except for the systemic AL score, the Mayo staging system and NT-proBNP <152 ng/L)		**Multivariable D** (including as covariates all parameters except for the systemic AL score, the Mayo staging system and historical criteria)	
	**HR (95% CI)**	**p value**	**HR (95% CI)**	**p value**	**HR (95% CI)**	**p value**	**HR (95% CI)**	**p value**	**HR (95% CI)**	**p value**
Age, per year	1.018 (1.004–1.031)	**0.010**	1.02 (1.007-1.035)	**0.002**	1.02 (1.006-1.033)	**0.006**	1.02 (1.007-1.035)	**0.003**	1.02 (1.007-1.034)	**0.003**
NYHA class	Reference		Reference		Reference		Reference		Reference	
II	4.07 (2.19-7.55)	**<0.001**	2.81 (1.37-5.78)	**0.005**	3.08 (1.48-6.43)	**0.003**	3.11 (1.52-6.34)	**0.002**	2.90 (1.48-5.70)	**0.002**
III	13.94 (7.33-26.50)	**<0.001**	8.70 (4.06- 18.64)	**<0.001**	8.71 (3.93- 19.29)	**<0.001**	10.06 (4.75-21.30)	**<0.001**	9.40 (4.60-19.19)	**<0.001**
IV	36.10 (16.38-79-70)	**<0.001**	20.52 (8.51-49.49)	**<0.001**	18.26 (7.06-47.20)	**<0.001**	22.79 (9.53-54.50)	**<0.001**	21.23 (9.02-49.97)	**<0.001**
Historical Criteria	5.29 (2.71-10.32)	**<0.001**	0.96 (0.35-2.62)	0.93			1.05 (0.39-2.79)	0.92		
NT-proBNP <152 ng/L	0.09 (0.02-0.39)	**<0.001**							0.00 (0.0-0.0)	0.95
Mayo Stage	Reference				Reference					
II	2.33 (1.24-4.38)	**0.008**			0.74 (0.31-1.75)	0.49				
IIIa	5.30 (2.90-9.70)	**<0.001**			0.97 (0.40-2.34)	0.94				
IIIb	12.03 (6.47-22.40)	**<0.001**			1.15 (0.45-2.91)	0.76				
"Systemic AL score"	Reference		Reference							
Possible	3.02 (1.30-6.98)	**0.010**	1.32 (0.49-3.60)	0.58						
Typical	7.83 (3.46-17.71)	**<0.001**	1.81 (0.65-5.03)	0.23						
ECV >0.40	4.20 (2.93-6.02)	**<.0.001**	1.64 (1.05-2.57)	**0.031**	1.82 (1.18-2.81)	**0.006**	1.92 (1.26-2.93)	**0.002**	1.90 (1.26-2.88)	**0.002**

In order to avoid statistical coupling of variables, separate multivariable models were performed that excluded coupled parameters (e.g., four separate models were created for systemic AL score, Mayo staging system, NT-proBNP <152 ng/L and historical criteria). For all the variables, the variable inflation factor was <2 suggesting that multicollinearity was not an issue.

AL, light chain; CI, confidence interval; ECV, extracellular volume; HR, hazard ratio; NYHA, New York Heart Association.

© 2024 Porcari A et al. *JAMA Cardiology.*

eTable 4. Multivariable Cox regression analyses for all-cause mortality according to different criteria of cardiac involvement in the landmark cohort at 6 months

**Variables**	**Multivariable A** (including as covariates all parameters except for the Mayo staging system and NT-proBNP <152 ng/L)		**Multivariable B** (including as covariates all parameters except for the systemic AL score and NT-proBNP <152 ng/L)		**Multivariable C** (including as covariates all parameters except for the systemic AL score, the Mayo staging system and NT-proBNP <152 ng/L)		**Multivariable D** (including as covariates all parameters except for the systemic AL score, the Mayo staging system and historical criteria)	
	**HR (95% CI)**	**p value**	**HR (95% CI)**	**p value**	**HR (95% CI)**	**p value**	**HR (95% CI)**	**p value**
Age, per year	1.03 (1.00-1.06)	0.051	1.02 (0.99-1.05)	0.08	1.03 (0.99-1.06)	0.054	1.03 (1.00-1.06)	**0.050**
NYHA class	Reference		Reference		Reference		Reference	
II	1.02 (0.36-2.87)	0.96	1.08 (0.38-3.07)	0.88	1.11 (0.40-3.07)	0.83	1.07 (0.41-2.75)	0.89
III	3.39 (1.06- 10.81)	**0.04**	3.19 (0.94- 10.83)	*0.06*	3.78 (1.22-11.69)	**0.021**	3.56 (1.23-10.37)	**0.019**
Historical Criteria	0.90 (0.16-4.95)	0.91			1.10 (0.22-5.43)	0.90		
NT-proBNP <152 ng/L							0 (0-0)	0.97
Mayo Stage			Reference					
II			0.95 (0.24-3.70)	0.94				
IIIa			1.17 (0.28-4.83)	0.82				
IIIb			1.44 (0.29-7.01)	0.65				
"Systemic AL score"	Reference							
Possible	1.62 (0.30-8.62)	0.57						
Typical	1.87 (0.32-10.79)	0.48						
ECV per each 10%	1.47 (1.04-2.07)	**0.029**	1.47 (1.05-2.05)	**0.025**	1.53 (1.12 – 2.10)	**0.008**	1.54 (1.13-2.10)	**0.006**

None of the patients in the landmark cohort at 6 months had NYHA class IV.
In order to avoid statistical coupling of variables, separate multivariable models were performed that excluded coupled parameters (e.g., four separate models were created for systemic AL score, Mayo staging system, NT-proBNP <152 ng/L and historical criteria). For all the variables, the variable inflation factor was <2 suggesting that multicollinearity was not an issue.

AL, light chain; CI, confidence interval; ECV, extracellular volume; HR, hazard ratio; NYHA, New York Heart Association.

© 2024 Porcari A et al. *JAMA Cardiology.*

eTable 5. Proposal for a new Staging System to redefine cardiac involvement in systemic AL amyloidosis

Mayo Clinic 2004 Staging System with European Modification			Treatment goal	Proposed New Staging System		ECV based individualised treatment goals s
Biomarker, cut-off	Stages			Stages	ECV, cut-off	
• cTnT ≥ 0.035 µg/L **or** cTnI ≥ 0.1 µg/L<br>• NT-proBNP ≥ 332 ng/L	I	Both < cut-offs	Deep and rapid HR	I	ECV < 0.30	HR at 1 month: does not impact survival<br>HR at 6 months: VGPR or better
	II	One > cut-off		II	ECV 0.31-0.40	HR at 1 month: does not impact survival<br>HR at 6 months: VGPR or better
	III	IIIa: Both > cut-offs<br>IIIb: NT-proBNP > 8.500 ng/L		III	ECV 0.41 – 0.50	HR at 1 month: at least VGPR<br>HR at 6 months: CR
	-	-		IV	ECV > 0.50	HR at 1 month: CR<br>HR at 6 months: CR

Legend: AL, Light Chain; cTnI, cardiac Troponin I; cTnT, cardiac Troponin T; ECV, Extracellular Volume, HR, Hematological Response.

© 2024 Porcari A et al. *JAMA Cardiology.*

SUPPLEMENTAL FIGURES

eFigure 1. Diagram of the study

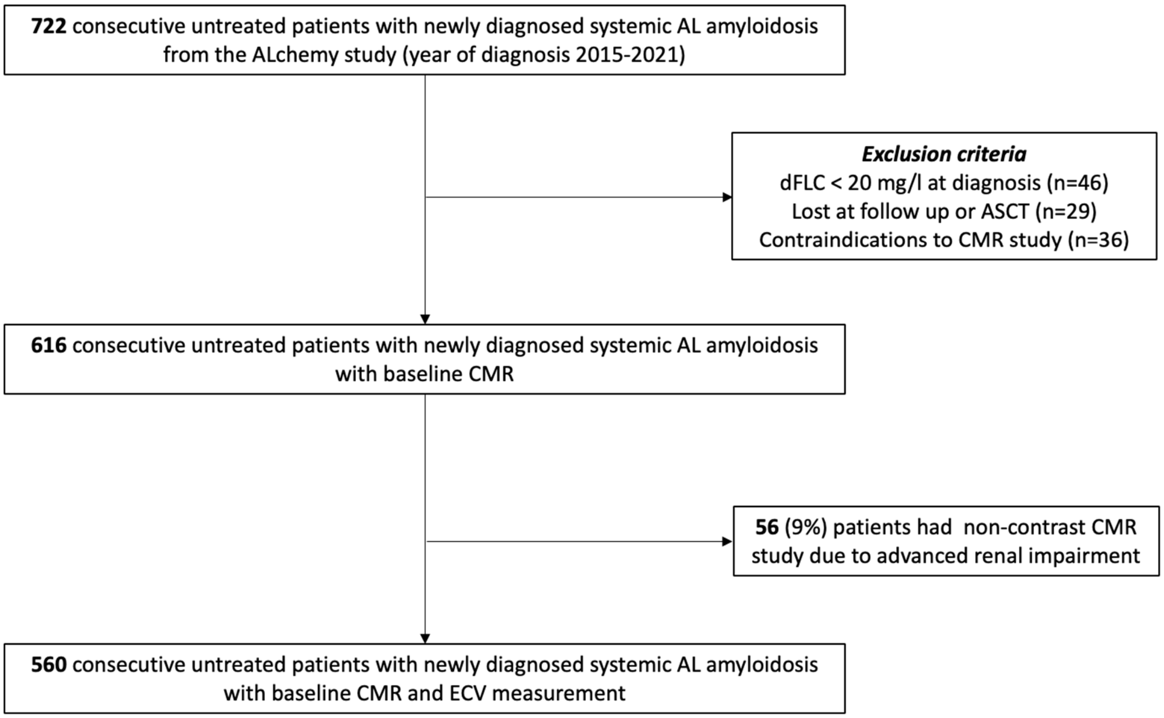

AL amyloidosis, systemic immunoglobulin light chain amyloidosis; ASCT, autologous stem cell transplant; CMR, cardiac magnetic resonance; dFLCs, difference in free light chains; ECV, extracellular volume.

© 2024 Porcari A et al. *JAMA Cardiology.*

eFigure 2. Distribution of cardiac amyloid load measured by ECV values using different criteria of cardiac involvement

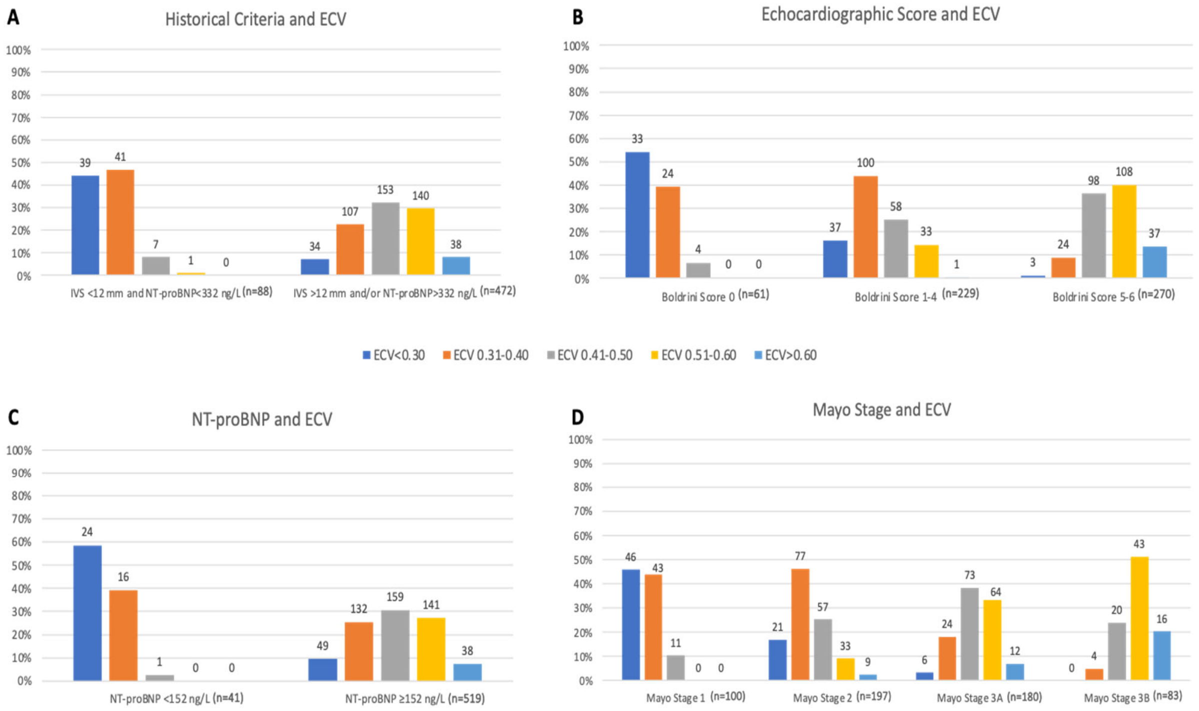

The number of patients is shown above each bar.

AL, light chain amyloidosis; ECV, extracellular volume; IVS, interventricular septum, NT-proBNP, N-terminal pro-brain natriuretic peptide.

© 2024 Porcari A et al. *JAMA Cardiology.*

eFigure 3. Kaplan-Meier analysis demonstrating the impact of baseline ECV mapping on overall survival among patients with AL amyloidosis. Legend:

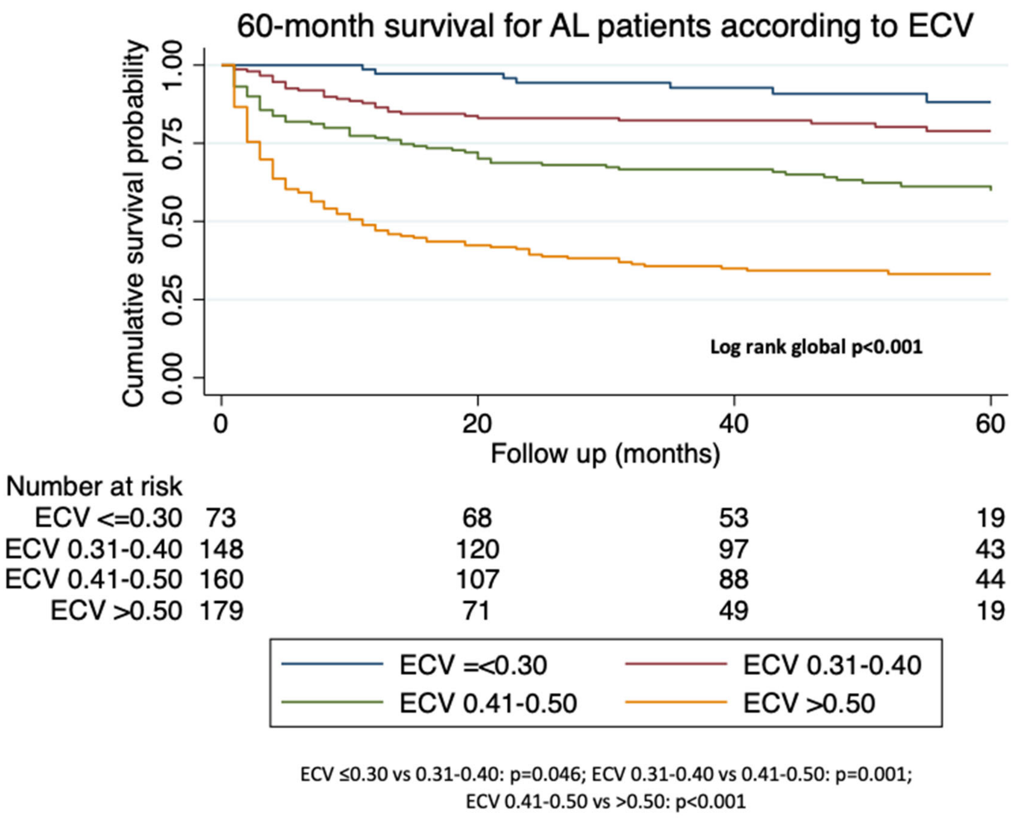

AL, light chain amyloidosis; ECV, extracellular volume.

© 2024 Porcari A et al. *JAMA Cardiology.*

eFigure 4. Association of early response (1-month) and survival stratified by baseline Mayo stages

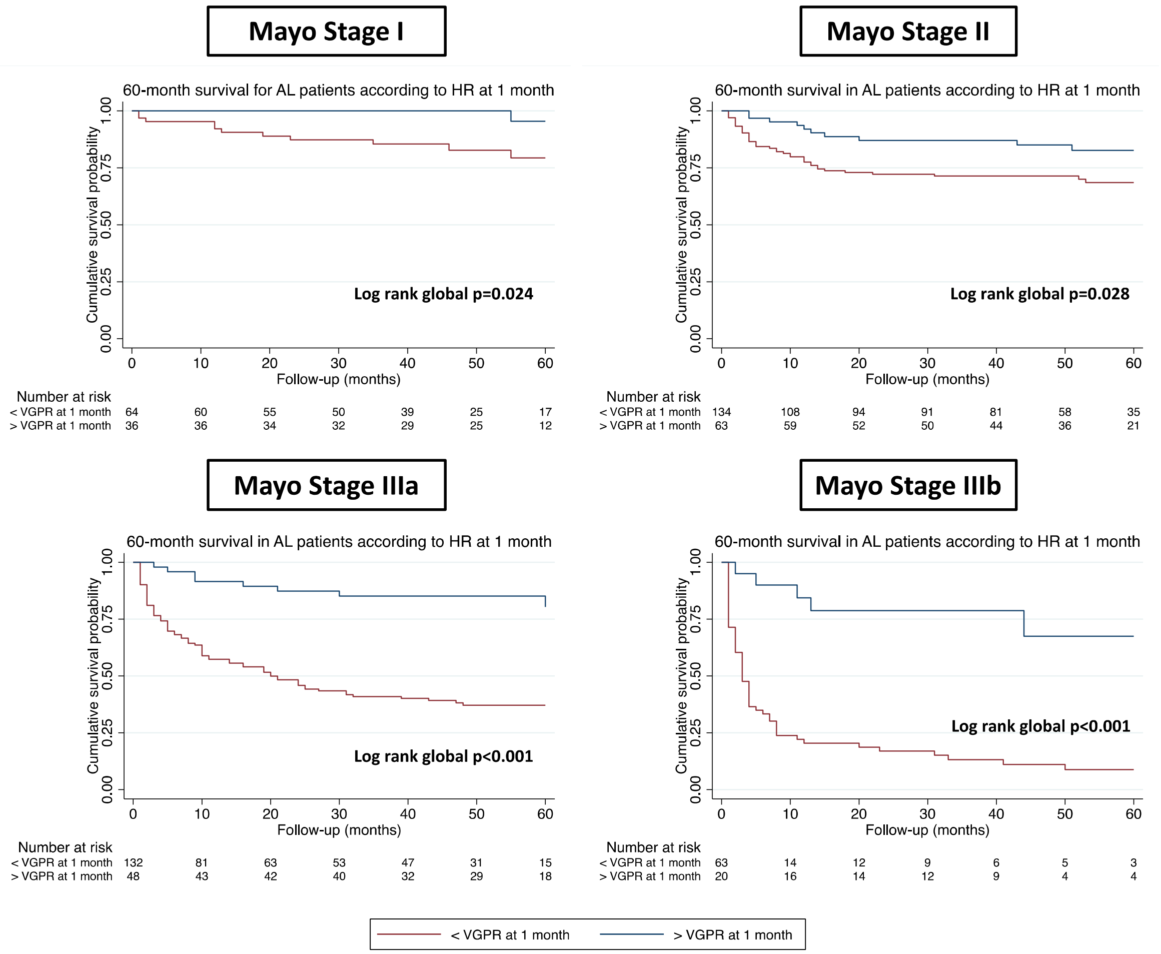

AL, light chain amyloidosis; HR, haematological response; VGPR, very good partial response.

© 2024 Porcari A et al. *JAMA Cardiology.*

eFigure 5. Association of deep response at 6 months and survival stratified by baseline ECV in AL patients with NR/PR at 1 month

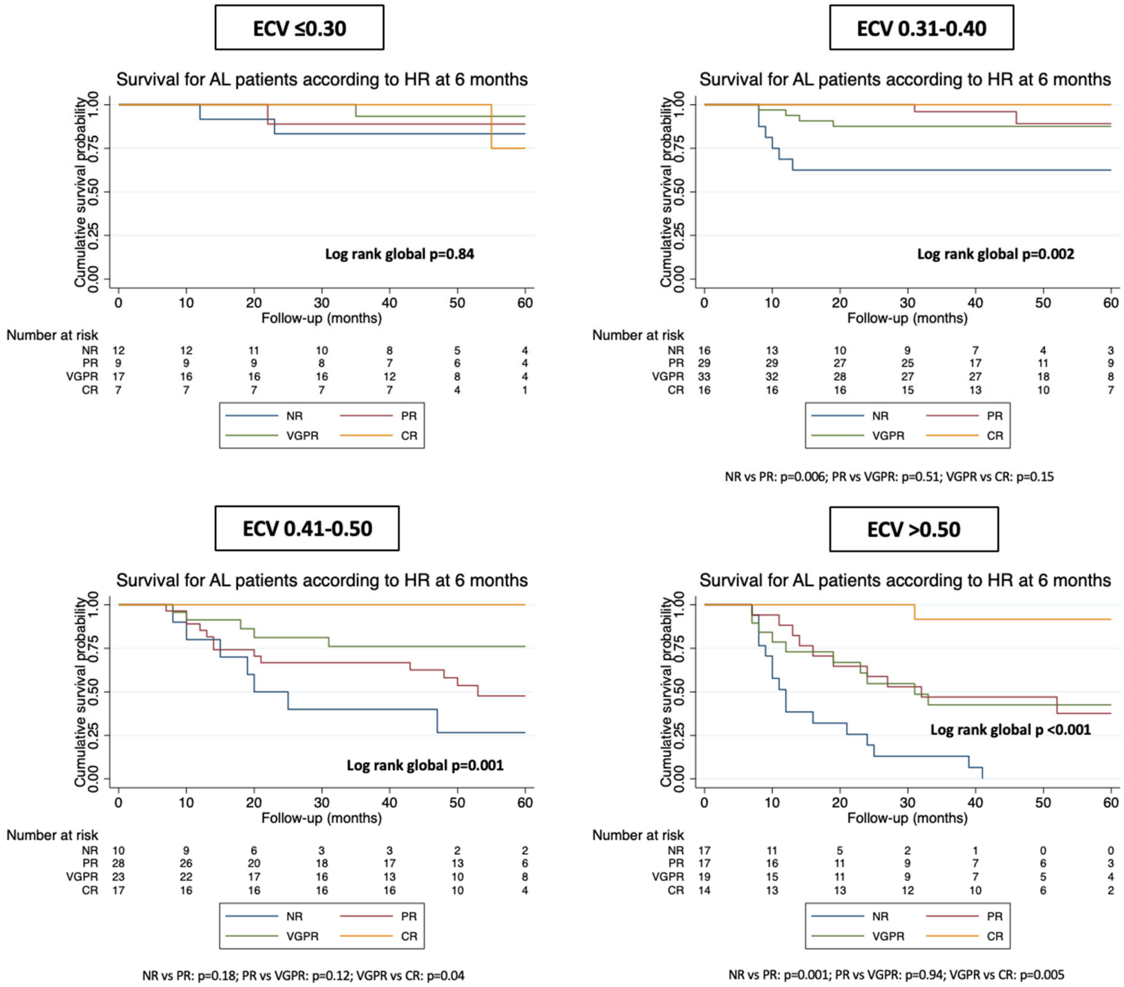

P values for inter-group comparison are shown only for subgroups with global p values <0.05.

CR, complete response; ECV, extracellular volume; HR, haematological response; NR, no response; PR, partial response; VGPR, very good partial response.

© 2024 Porcari A et al. *JAMA Cardiology.*
