# Supplementary material for: Redefining Cardiac Involvement and Targets of Treatment in Systemic Immunoglobulin AL Amyloidosis
Source: JAMA Cardiol. 2024 Aug 21;9(11):982–9. doi: 10.1001/jamacardio.2024.2555 (PMC11339700; doi:10.1001/jamacardio.2024.2555)
Supplement: Supplement 2. — Data sharing statement [file jamacardiol-e242555-s002.pdf]

Data Sharing Statement

Porcari. Redefining Cardiac Involvement and Targets of Treatment in Systemic Immunoglobulin AL Amyloidosis. *JAMA Cardiol*. Published August 21, 2024. doi:10.1001/jamacardio.2024.2555

Data
Data available: No

Additional Information
Explanation for why data not available: The data underlying this article cannot be shared publicly because of the privacy of individuals who participated in the study. The data will be shared on reasonable request to the corresponding author.
